# Human papillomavirus infection-related cancer risk for kidney transplant recipients during adult life can be reduced by vaccination during childhood and adolescence

**DOI:** 10.3389/fped.2022.1057454

**Published:** 2022-11-30

**Authors:** Corina Nailescu, Marcia L. Shew

**Affiliations:** Department of Pediatrics, Riley Hospital for Children, Indiana University, Indianapolis, IN, United States

**Keywords:** human pappillomavirus, vaccination, kidney transplantation, malignancy, adolescence

## Abstract

Malignancies are found between the first three reasons of mortality in pediatric and adult kidney transplant recipients, who overall have disproportionately higher rates of cancer compared to the general population, including human papillomavirus (HPV)-related genital, anal and oropharynx region cancers. Therefore, preventing HPV in this patient population is extremely important. HPV-vaccine was demonstrated to prevent HPV infection in individuals with intact immune systems. In addition, recent data reported less precancerous HPV lesions and cervical cancers with use of HPV vaccine. Since HPV is a sexually transmitted virus that is typically acquired shortly after the onset of sexual activity, it is best to administer the HPV vaccine immunization prior to the onset of sexual activity. This article reviews the epidemiology and pathophysiology of the HPV infection, as well as its role in the development of HPV-related pre-cancerous lesions and cancers in both general population and kidney transplant recipients. The focus is on the most effective primary prophylactic strategy, which is the HPV vaccination. The particularities of HPV vaccination strategies in kidney transplant recipients are compared to the general population. In addition, the article analyzes the various causes of suboptimal HPV immunization rates in kidney transplant candidates and recipients and discusses vaccination optimization strategies that can be applied during childhood and adolescence to reduce the burden of HPV-related disease states and cancer among adult kidney transplant recipients.

## Introduction

Cancers in general are between the first three reasons of death in adult solid organ transplant (SOT) recipients, including kidney transplant (KT) recipients (1). It is disturbing to know that by 30 years following transplantation, 26%–41% of SOT recipients who had received their transplants as children, would have developed some form of malignancy (2). Overall, the cancer rate in adults who received a KT as children was six times higher compared to general population (3). As an example, female KT recipients have a 14-fold increased risk of cervical cancer and a 50-fold increased risk of vulvar cancer, male KT recipients have an increased risk for penile cancers and both female and male KT recipients have a 100-fold increased risk for anal cancers (4–7). The common denominator for all these cancer types is the infection with different types of human papillomavirus (HPV) (8).

Understanding that adult KT patients carry a high-risk for cancers, including HPV-related ones, and that the treatment for these diseases can negatively impact the graft viability and life expectancy**,** medical professionals need to get an early start with the most efficacious preventive strategies during childhood and adolescence (4). This article will delineate the most efficient primary prevention strategy, the HPV vaccination, which applied by pediatric professionals during childhood and adolescence has been proven to reduce the risk for HPV-associated malignancies for KT recipients during their adult life (9, 10).

## HPV: epidemiology

HPV is known as the most prevalent sexually transmitted infection (11). Worldwide, the estimated lifetime probability of being infected is greater than 80% for women and 90% for men (12). Certain types, including HPV 16, −18, −31, −33, −35, −39, −45, −51, −52, −56, −58 and −59, are responsible for greater than 95% of HPV-related cancers (4, 13). HPV has been identified as a cause for cervical, vaginal, vulvar, penile, anal, and oropharyngeal cancers in >91%, 75%, 69%, 63%, 91% and 70%, respectively (4, 14). Of all the HPV-related malignancies, cervical carcinoma, which is linked to HPV in over 90% of the cases, deserves a special consideration, as it is the second most common cancer and cause of cancer death in women at international level (4, 14). HPV 16 carries the highest risk for development of invasive cervical cancer (15). Specifically in adult women KT recipients, cervical cytology was diagnosed as atypical in 25.8% of cases, while the cervical presence of HPV was detected in 22.5% of the patients (16). HPV 16, and to a smaller degree HPV 18, are also the root cause for most oropharyngeal and anal cancers (17, 18). Specifically, adult KT recipients had almost three-fold higher adjusted odds of having anal high-risk HPV lesions compared to healthy controls (19). Of note, certain non-oncogenic HPV types such as 6 and 11 are associated with genital warts and respiratory papillomatosis (20). Specifically, adult KT recipients had a higher hazard of genital warts compared to the general population (HR = 3.3; 95% confidence interval, 2.76–3.93) (21). While non-oncogenic, these HPV types can nevertheless lead to serious and difficult to treat disease in the face of immunosuppression (22, 23).

## HPV: natural history

The natural history of HPV infection is better understood in the cervix, as it is thought that women in general encounter one or multiple types of HPV infections within the first two years after their sexual debut (24, 25). Luckily, many of these early HPV cervical infections become non-detectable within two years (24). Whether this non-detection represents total clearance of the virus or suppression in a latent stage is generally unknown (26). If high-risk HPV infections are persistent, they can progress to precancerous lesions known as cervical intraepithelial neoplasia (CIN) (graded as 1, 2 and 3) (27). Such pre-cancerous lesions can over time can progress to invasive malignant lesions (27). Risk factors associated with acquiring new HPV infections and progressing to CIN or even cancer include young age sexual debut, tobacco use, and higher number of sex partners, as well as immunocompromised state (28). Indeed, immunosuppression has been associated with poor cell mediated viral clearance leading to increased infection rates, persistent infections, accelerated reactivation from the latent phase and accelerated progression of precancerous disease to cancer (29–33).

By contrast, the natural history of HPV infection in the anal and oropharyngeal regions is less clear. However, similar to cervical dysplasia, anal cancer has its cytologic precursors that may be treated early to prevent progression (34). Both anal and oral cancers carry similar risk factors for acquisition of the virus (34). In studies specific to KT, individual findings have shown an increased presence of HPV (compared to non-immunosuppressed populations) in the oral cavity (35), higher prevalence of genital warts (21) and increased risk of HPV infections and cancer in the anal region (36, 37).

## HPV vaccination

Prevention strategies have been developed to protect the general population from the HPV-related cancers. As primary prevention, the development and clinical use of the HPV vaccine has allowed a very effective intervention against HPV- related cancers in the general population (38). Secondary prevention, involving the detection of early cancer mostly using cervical cytology (Pap smear methodology), as well as HPV testing, has also been successfully used in the general population (39). Unfortunately, applying such primary and secondary HPV prevention strategies to immunosuppressed patients, including KT recipients, has been less than optimal. This article should help KT professionals understand the HPV vaccination particularities in the patient population they are carrying for, in comparison to the general population, thus hopefully resulting in improved immunization rates.

Three types of HPV vaccines are available. The quadrivalent HPV vaccine is effective against types 6, 11, 16 and 18 and it was approved in 2006 for female patients age 9–26 and in 2009 for males age 9–26, as well. The bivalent vaccine, effective against HPV types 16 & 18 was approved only for female patients age 10–25 in 2009. The 9-valent vaccine, which is effective against HPV types 6, 11, 16, 18, 31, 33, 45, 52 and 58 was licensed for both female and male patients age 9–26 in 2014 (40). In the United States only the 9-valent vaccine is currently available.

### HPV vaccination in the general population

#### Effectiveness

HPV vaccines are extremely efficacious in preventing persistent HPV infections with oncogenic types, as well as the development of premalignant and malignant HPV-related pathology (41). Multiple studies have shown a reduction in abnormal cervical precancerous lesions, cervical cancers and anal cancers secondary to the use of HPV vaccine (38, 42, 43). For example, after the introduction of the HPV vaccine in 2006, during 2008–2015, the rates of CIN2+ in the state of Connecticut declined by 30%–74% among women aged 21–26 years, with greater declines observed in the younger women (42). In regards to anal intraepithelial neoplasia, the efficacy of the quadrivalent HPV vaccine in preventing it was estimated to be 50.3% (95% confidence interval 25.7–67.2) in the intention-to-treat population and 77.5% (95% confidence interval 39.6–93.3) in the per-protocol efficacy polupation (43). In addition, there is also emerging evidence of possible oropharyngeal cancer prevention with the use of HPV vaccine (44). It should be noted that the vaccine is approved to prevent HPV-related cervical and anal precancer and cancer, but it is not approved to prevent HPV-related oral cancer (45).

#### Dosing

When the vaccine series is initiated before age 15, the regimen consists of two doses. The second dose should be administered six to twelve months after the initial one. For patients who accidentally receive the two vaccine doses less than five months apart, a third dose of the HPV vaccine is recommended (46).

When the vaccine series is initiated between age 15 and 26, the regimen consists of three doses. The recommended schedule for this three-dose regimen is to be administered at time zero, at one to two months and at six months after the initial one. The vaccine is not routinely offered after age 26 (40) (Table 1).

**Table 1:** Dosing and timing recommendations for HPV vaccine administration.

Age	Healthy Individuals	KT Recipients*
9 to 15	Two doses of HPV vaccine at month:	Three doses of HPV vaccine at month:
	−0	−0
	−6–12	−1–2
		−6
15 to 26	Three doses of HPV vaccine at month:	Three doses of HPV vaccine at month:
	−0	−0
	−1–2	−1–2
	−6	−6
26–45	Not routinely offered	Should be considered**

* For HPV vaccine administration in KT recipients it is recommended to wait 3–6 months after transplant, similar to other non-live vaccines except influenza.

** HPV vaccination is safe for adults aged 26 through 45 years, however population benefit is minimal; however, KT recipients who are not adequately vaccinated may be at risk for new HPV infection and therefore might benefit from vaccination in this age range. Therefore, should be considered in individual cases as shared clinical decision-making between patient and provider.

#### Timing

The HPV vaccine is effective in preventing infection, but not in clearing previously acquired infections (46, 47). Since HPV is a sexually transmitted infection typically acquired shortly after the sexual debut (48), HPV vaccination is optimal when administered before the onset of sexual activity (4). In the United States, 5% to 25% of males residing in metropolitan areas reported having sexual intercourse before age 13 (49). The data on such sexual experiences of adolescents, as well as the known robust immune responses that adolescents have compared to older individuals, formed the basis for the recommendation of administering the HPV vaccine at 11 to 12 years of age (4, 46). However, the HPV vaccine can be given as early as nine years of age (46) with catch-up vaccination through age 26.

#### Short-term response to HPV-vaccination

In healthy populations, the seroconversion rates are 95%–100% after vaccination (50, 51). This vaccination-induced immune response is clearly much more robust compared to the natural infection, as in one study only 54%–69% of women with incident HPV 16 or 18 infections had antibodies (52).

#### Long-term persistence of immunity

Data from a ten-year post-bivalent HPV vaccination follow-up European study performed in immunocompetent individuals found that seropositivity rates remained relatively high: 100% for HPV-16 and 99.2% for HPV-18; using mathematical models, immunity was predicted to remain above natural infection levels for at least 30 years (53). In addition, sustainability of neutralizing antibodies induced by HPV vaccines in a combined follow-up analysis of data from two randomized, double-blinded, multicenter, phase 3 trials showed that HPV vaccine-induced seroprotection can be maintained up to 12 years (54).

### HPV vaccination in KT recipients

#### Effectiveness

The studies evaluating the vaccine in reducing HPV disease endpoints in immunosuppressed individuals, including human immunodeficiency virus (HIV) patients and SOT recipients, continue to be limited, but emerging. For example, the quadrivalent HPV vaccine has been recently shown to reduce HPV infections and genital warts in HIV women (55, 56). There is also recent data demonstrating that in immunosuppressed individuals (HIV-infected, with a SOT history, including KT, or being on medications that suppress the immune system), HPV vaccination was associated with the development of less CIN2 + precancerous lesions (9, 10). In addition, there are several case reports describing the resolution of genital and non-genital difficult-to-treat recalcitrant warts after HPV vaccine administration (57, 58).

#### Dosing

The dosing for KT recipients, which actually applies to all SOT recipients, is slightly different than for the general population. In essence, while healthy individuals less than 15 need only two doses of the vaccine, KT recipients should always receive three doses, regardless of their age (59). Furthermore, while in healthy individuals the vaccine is not routinely offered after age 26, in KT recipients it can be taken into consideration (4) (24), (Table 1).

#### Timing

Timing of the HPV vaccine for KT recipients should be as recommended for the general population. It is optimal to vaccinate whenever possible prior to transplantation, during the last stages of chronic kidney disease (CKD) or while on dialysis, as it has been shown that after transplantation the vaccine responses are reduced (4, 60).

#### Short-term response to HPV-vaccination

The HPV vaccines are efficacious in SOT recipients, including KT, but nevertheless the immune response appears to be reduced compared to the general population (4). The very first study of the HPV vaccine in SOT recipients that was done in 47 adults, both females and males, showed lower seroconversion rates: 63.2%, 68.4%, 63.2% and 52.6% for HPV 6, 11, 16 and 18, respectively, compared to historical healthy controls (61). More recently, a Belgian study assessed the 9-valent HPV vaccine response in adults with kidney, lung and heart transplants and found lower seroconversion rates, ranging from 46% for HPV45 to 72% for HPV58 (62). The very first pediatric study included 57 age 9–21 subjects with KT and showed that seropositivity was significantly lower (62.5%, 50.0%, 75.0% and 50.0% for HPV 6, 11, 16 and 18, respectively), compared to patients with advanced CKD (63). Our multicenter study included 72 patients, females and males age 10–16 with KT and reported that the KT recipients were significantly lower in terms of seroconverting (72.4%, 69.0%, 89.7% and 62.1%, for HPV 6, 11, 16 and 18, respectively), compared to patients with CKD (100%, 100%, 100% and 94.4%) (60). The evidence points out that it is optimal to vaccinate whenever possible before transplantation, during the last stages of CKD or on dialysis (4). If HPV vaccine was not administered before transplant, it is still recommended after, at least three to six months post-transplantation for the best optimal immune response given the circumstances (64).

#### Long-term persistence of immunity

Five years post-HPV vaccination in SOT recipients, seropositivity rates dropped from 100% immediately post-vaccination to 94.6% for HPV-16 and from 91.2% to 78.4% for HPV-18 in a recent Australian study (65). This study raised the question of the utility of revaccinating SOT recipients after a number of years. It has been shown that in immunocompetent individuals, revaccination with the HPV vaccine resulted in an increase in antibody titers and anamnestic responses from B cell memory (66). To this date, re-vaccination with HPV vaccine has not been studied in immunosuppressed individuals and in addition, the exact neutralizing antibody titer needed to prevent infection is unknown, therefore the need for revaccination in SOT recipients has not been so far demonstrated (4).

## Discussion: strategies to improve the suboptimal HPV immunization rates in KT recipients

In a study of pediatric KT recipients from University of Minnesota followed long-term it was noted that at 20 years post-KT, 13% of the patients were suffering from a form of malignancy, while by 30 years post-KT, 26% (3). Despite the fact that KT recipients are known to have a high risk for malignancies, including HPV-associated malignancies, unfortunately they do not appear to be ideally protected by primary prevention: they end up either not immunized, or immunized beyond the recommended time (67). It is known that the HPV vaccine acceptance by either patients or caregivers has always been a problem even in healthy populations (68, 69). When it comes to KT patients, HPV vaccination status is even less optimal, likely because data on safety and efficacy has been scant until recently (67). However, recent studies provided more evidence that patients who are likely to need a KT in the future respond best to the HPV vaccine before transplant, while with CKD or on dialysis (60, 63). Since the leading causes of end-stage renal disease in children are congenital anomalies of the kidneys and the urinary tracts in which the kidney function diminishes slowly, pediatric nephrologists should be able to better plan a KT compared to their colleagues who care for adults when it comes to vaccinations (67). Therefore, the results of the recent studies brought forward new information to help pediatric medical professionals in their work to complete the HPV vaccine administration ideally prior to transplantation if possible (4). It is also to emphasize that pediatric nephrologists have dialysis as a temporary treatment option for their patients with end-stage renal disease, which allows them to best prepare their patients for a transplant, including establishing a complete vaccination status (4). In the case of SOT other than the KT, it is not always possible to plan so much in advance, therefore it is equally important for general pediatricians to make sure HPV vaccination happens promptly at the minimum vaccination age.

KT recipients appear to be vaccinated later than the recommended time (60). This is likely because these patients are chronically ill with CKD while awaiting transplantation and therefore there is less concern about sexual activity, as demonstrated in patients with other types of chronic diseases (60, 70). Surprisingly, a recent study of children with CKD showed that growth and sexual maturation are only slightly delayed compared to healthy subjects, which is contrary to what it was previously thought (4, 71), therefore it is important that they are administered the HPV vaccine without delays (60). In general, routine pediatric immunizations in KT candidates and recipients are more likely to be administered by their primary care providers, while vaccines that differ from the routine schedules, including the HPV, are more likely to be administered by transplant professionals, who are likely to be more familiar with dosing them appropriately (4, 67). Thus, it is important for both general pediatricians and pediatric transplant nephrologists to be aware of the immunization guidelines for KT candidates and recipients and stay in close communication, to ensure an ongoing fully immunized status for their patients (4).

In addition, vaccine hesitancy has been an even greater issue in successfully vaccinating children and adolescents, therefore general pediatricians and pediatric transplant nephrologists can help to overcome reluctance by educating their patients regarding to the safety profile and the benefits of the HPV vaccine (72). In order to improve HPV immunization in the general population, different interventions have been shown to be somewhat effective, including provider education, collaboration between physicians and pharmacists and systems changes (73, 74). In a recent study, such interventions modestly increased HPV vaccination rates from 60% preintervention to 69% postintervention (75). Another intervention showed more promising results, as parents who watched an educational video had 3-times greater odds of accepting that their children receive the HPV vaccine (76).

In conclusion, in order to optimally protect KT patients from acquiring HPV-associated cancers, pediatric transplant professionals should advocate that their patients get the HPV vaccine before transplantation, provided that they have reached age nine. If the KT occurs during the series, vaccination should be completed afterwards, resuming the series at least 3–6 months after transplant. If still unvaccinated post-transplant, medical professionals should recommend the HPV vaccine even after age 26. Further research will be needed to assess the effectiveness of the HPV vaccine when given before transplantation in preventing actual HPV-related pathology. Consideration is being given to administering the vaccine at an earlier than currently recommended age. If proven effective enough and safe, this could change the life of many patients, who otherwise would become transplanted without the HPV vaccine and therefore would miss the opportunity to mount a strong serologic response pre-transplant.
